# Serum NGAL, KIM-1, IL-18, L-FABP: new biomarkers in the diagnostics of acute kidney injury (AKI) following invasive cardiology procedures

**DOI:** 10.1007/s11255-020-02530-x

**Published:** 2020-06-17

**Authors:** Magdalena Zdziechowska, Anna Gluba-Brzózka, Adam R. Poliwczak, Beata Franczyk, Michał Kidawa, Marzenna Zielinska, Jacek Rysz

**Affiliations:** 1 Department of Nephrology, Hypertension and Family Medicine, Medical University of Lodz, Zeromskiego 113, 90-549 Lodz, Poland; 2 Department of Human Physiology, Chair of Experimental and Clinical Physiology, Medical University of Lodz, Lodz, Poland; 3 Department of Intensive Cardiac Therapy, Central Teaching Hospital of the Medical University of Lodz, Lodz, Poland

**Keywords:** NGAL, L-FABP, KIM-1, IL-18, Acute kidney injury, Contrast-induced, Biomarkers

## Abstract

**Purpose:**

The aim of this study was to assess the levels of selected markers in patients who underwent planned or emergency coronary angiography and to examine if they correlated with the occurrence of AKI.

**Methods:**

The study included 52 patients who underwent planned or emergency coronary angiography and received contrast agent. Serum levels of markers (NGAL, L-FABP, KIM-1, IL-18) were analyzed in all patients using ELISA tests, at baseline, after 24 and 72 h from angiography.

**Results:**

9.62% of patients developed CI-AKI. No significant differences were observed between markers levels in patients who developed CI-AKI and those who did not. After 24 h, serum levels of IL-18 were higher in patients with CI-AKI, however, this difference was on the verge of significance. Increase in serum NGAL, KIM-1 and IL-18 was observed after 24 h. Serum levels of biomarkers were insignificantly higher in group with CI-AKI. Significant changes in levels in time (baseline vs. 24 h vs. 72 h) were observed only for NGAL [157.9 (92.4–221.0) vs. 201.8 (156.5–299.9) vs. 118.5 (73.4–198.7); *p* < 0.0001]. No significant correlations were observed between the decrease in eGFR or the increase in creatinine and biomarkers level.

**Conclusion:**

Obtained results do not allow for the indication of efficient AKI biomarkers. Their further validation in large studies of CI-AKI patients is required.

## Introduction

Acute kidney damage is a clinical syndrome that develops as a result of sudden impairment of kidney function [1]. It is characterized by a wide spectrum of disorders ranging from a temporary increase in the concentration of biological markers, impaired renal function (elevated creatinine levels, decrease in GFR) to severe metabolic and clinical disorders, or even the necessity to undergo renal replacement therapy. Acute kidney injury occurring shortly after the intravenous injection of iodinated contrast material (CM) includes: post-contrast AKI which may or may not be directly caused by the contrast material and contrast-induced AKI (CI-AKI), previously called "contrast-induced nephropathy” (CIN), which can be causally linked to contrast material administration. CI-AKI has been observed after the use of radiocontrast media for coronary angiography (CAG) with or without percutaneous coronary intervention (PCI) [2]. The reported incidence of CIN in high-risk patients following CAG varies from 10 to 20% [3]. CI-AKI risk in post-PCI patients could be related either to the patient (advanced age, concomitant diseases, dehydration, and concomitant use of other nephrotoxic drugs) or procedure related (intra-arterial route of administration, the use of high osmolar CM, repeated exposure to contrast within 48 h, volume of contrast used, etc.) [4]. Among risk factors for the development of acute kidney damage there are: age > 70 years, diabetes mellitus, chronic kidney disease, circulatory failure, atrial fibrillation, gout, infections, surgery, some drugs (NSAIDs, diuretics, aminoglycosides and vancomycin) [5]. The prevalence of CI-AKI in persons with normal renal function is low (0–5%), but it increases along with the deterioration of kidney function—in patients with moderate impairment of renal function (GFR 45–60 ml/min) it reaches about 10–30%, in patients with severe renal impairment (GFR < 45 ml/min) it is less than 50%, while in diabetic nephropathy it exceeds 50% [6]. Some former studies reported high risk of CI-AKI in general population, however, according to more recent studies its incidence is about 0.8–1.7%. The discrepancies in the incidence of CI-AKI in many studies may be partly explained by the fact that small rises of serum creatinine are quite common in sick patients, even in those not receiving contrast [7, 8] and also by the current use of lower doses of contrast, improved technologies as well as risk stratification of patients which enables the avoidance of contrast administration in the most risky patients. Contrast-induced nephropathy involves acute kidney damage with acute tubular necrosis sometimes leading to end-stage renal disease [9]. The Kidney Disease Improving Global Outcomes (KDIGO) working group suggests that AKI should be diagnosed when serum creatinine level has increased by at least 0.3 mg/dl (26.5 μmol/l) over the baseline value within 48 h after the exposure to contrast medium, or it has increased 1.5 times or more over the baseline value within 7 days after the exposure to contrast medium, or a urinary volume of less than 0.5 ml/kg/h that persists for at least 6 h after exposure is observed [10]. Moreover, other causes for AKI such as atheroemboli, among others should be excluded [7].

Acute kidney damage increases patients’ morbidity and mortality, prolongs hospitalization and enhances the risk of chronic kidney disease and cardiovascular events [6]. Due to the seriousness of CI-AKI prognosis, it should be recognized as soon as possible, effectively treated, and preferably prevented. The monitoring of renal function on the basis of creatinine and GFR in the assessment of contrast-induced nephropathy, is not always sufficient. Apart from the determining of serum creatinine (which is a late marker of kidney function, not its damage), new biomarkers of acute kidney damage are helpful in the diagnosis. Neutrophil gelatinase-associated lipocalin (NGAL), kidney injury molecule-1 (KIM-1), interleukin-18 (IL-18) and liver fatty acid-binding protein (L-FABP) have been suggested to be early markers of acute kidney injury, which effectiveness should be confirmed in studies [3, 4, 11]. NGAL is a glycoprotein produced by neutrophils and epithelial cells of various organs [12]. This protein is released from the proximal nephron tubule cells into the bloodstream following ischemia and nephrotoxic damage [13]. NGAL seems to be a good early marker in the diagnosis of acute kidney damage in patients undergoing PCI because the increase in its concentration occurs significantly earlier than changes in creatinine levels [14]. The peak of NGAL level in serum was observed 2 h after PCI [15].

KIM-1 is a type 1 transmembrane protein which expression is significantly increased in the proximal tubule in the ischemic kidney [16, 17]. KIM-1 is a promising factor in the diagnosis of acute kidney damage with a predictive value in patients undergoing cardiac surgery. This is the first biomarker that specifically reflects proximal renal tubular damage [16, 17].

IL-18—is a pro-inflammatory cytokine which activity increases in the course of inflammatory processes. It is a mediator of tissue damage caused by hypoxia [18]. Urine IL-18 is an early, fast and inexpensive marker allowing the detection of early kidney damage caused by ischemia or nephrotoxins [4]. However, a little is known about the potential of serum IL-18 as AKI biomarker.

L-FABP—liver fatty acid binding protein—is a newly discovered biomarker for early diagnosis of acute conditions [19]. In acute renal damage caused by contrast agents during PCI, a significant increase in L-FABP excretion has been demonstrated after 1, 2 and 14 h from the procedure in patients who developed contrast nephropathy [20].

The aim of this study was to assess the levels of selected markers in patients who underwent planned or emergency coronary angiography and to correlate it with possible occurrence of CI-AKI.

## Materials and methods

This study included 52 patients from the Med Pro Cardiological Center of Zgierz and from the Department of Intensive Cardiac Therapy, Central Teaching Hospital of the Medical University of Lodz, who underwent planned or emergency coronary angiography and received contrast agent. The study was approved by the Bioethics Committee of the Medical University of Lodz (No. RNN/26/18/KE of 16.01.2017) and conducted according to the principles expressed in the Declaration of Helsinki. All subjects enrolled in this study were informed about its purpose and methodology and signed an informed consent form. Exclusion criteria were as follows: state after kidney transplantation, haemoglobin < 8 g/dl, active cancer, active hepatitis B or C, mental disease, alcoholism, HIV infection or other immune disorders and lack of consent to participate in the study. A complete medical history was obtained from all participants. The levels of selected markers were analyzed in blood of all patients involved in the study. In the present study, serum levels of selected biomarkers of proteins: KIM-1 (Cloud-Clone Corp, no. SEA785Hu), IL-18 (BioVendor, No. RAF143R), Lipocalin-2/NGAL (BioVendor, No. RD191102200R), L-FABP (MyBioSource, No. MBS017865) were determined using ELISA method according to the manufacturer’s instructions. Serum creatinine, NGAL, KIM-1, IL-18, L-FABP were measured at baseline and after 24 and 72 h after angiography. Additional measures for preventing CI-AKI were individualized according to the recommendations of attending physician. Estimated glomerular filtration rate (eGFR) was calculated using simplified 4-variable Modification of Diet in Renal Disease (MDRD) formula:

eGFR = 186 × (serum creatinine)^−1.154^ × (age)^−0.203^ × [0.742 if female] [21].

Contrast-induced AKI (CI-AKI) has been diagnosed in patients in whom a 25% creatinine rise within 48 h was observed [22]. Heart failure was diagnosed based upon available medical history, or typical signs and symptoms as well as on objective evidence of structural or functional abnormality of the heart (ESC HF Guidelines 2016) [23].

### Statistical analysis

Data were presented as percentages or as median with interquartile range (median 25–75%) to account for the skewed nature of parameters given the small sample size. The Shapiro–Wilk test was used to verify normal distribution of variables and Levene test to analyze the homogeneity of variance. The *χ*^2^ test of independence was used for the analysis of discontinuous variables. Standard Student *t* test was utilized for the comparison of data showing no departures from normality, and the non-parametric Mann–Whitney *U* test for the remaining variables. Friedman nonparametric test was used to compare levels of selected markers in different time points. Significance level *p* < 0.05 was considered as statistically significant. Statistical analyses were performed using GraphPad Prism7, GraphPad Software, San Diego, CA, USA.

## Results

Table 1 presents baseline characteristics of all patients enrolled into this study.

**Table 1:** Baseline characteristics of patients

Total sample *n* = 52	
Age (years)	66.4 ± 8.7
Male *n* (%)	31 (60%)
BMI	29.2 ± 4.1
Hypertension *n* (%)	45 (87%)
Diabetes mellitus *n* (%)	15 (29%)
Heart failure	26 (50%)
Atrial fibrillation	9 (17%)
Hypercholesterolemia *n* (%)	22 (42%)
Planned coronary angiography *n* (%)	12 (23%)
Emergency coronary angiography *n* (%)	40 (77%)
Indication for PCI at index admission *n* (%)	
STEMI	1 (2%)
NSTEMI	9 (17%)
UA	35 (67%)
Arterial stenosis *n* (%)	19 (37%)
Serum creatinine at admission (μmol/L)	81.0 ± 20.0
Mean eGFR at admission (mL/min/1.73 m^2^)	83.4 ± 20.7
eGFR at admission < 60 mL/min/1.73 m^2^	7 (13%)
Mean contrast-medium volume	111.7 ± 88.7
Serum NGAL at admission (ng/mL)	201.8 (156.5–299.9)
Serum KIM-1 at admission (ng/mL)	130.2 (42.4–341.9)
Serum IL-18 at admission (ng/mL)	261.6 (241.7–365.7)
Serum L-FABP at admission (ng/mL)	4.7 (2.7–5.0)

*STEMI *ST elevation myocardial infarction, *NSTEMI* non-ST-elevation myocardial infarction, *UA* unstable angina

Of the 52 recruited patients, 5 (9.6%) developed CI-AKI. Patients who developed CI-AKI were more likely to be older, to have STEMI or NSTEMI and higher GFR. Table 2 presents the comparison of characteristics of patients who developed CI-AKI and who did not.

**Table 2:** Baseline characteristics of patients who developed CI-AKI and those who did not

Demographic	Group of patients who developed CI-AKI (*n* = 5)	Group of patients who did not develop CI-AKI (*n* = 47)	*p* value
Male (*n*, %)	4 (80%)	27 (57%)	0.33
Age (years)	67.1 ± 8.8	60 ± 4.3	**0.05**
BMI	29.3 ± 2.2	29.1 ± 4.3	0.94
Hypertension (*n*, %)	5 (100%)	40 (85%)	0.81
Hypercholesterolemia (*n*, %)	1 (20%)	21 (45%)	0.56
DM (*n*, %)	3 (60%)	12 (26%)	0.27
Atrial fibrillation (*n*, %)	2 (40%)	7 (15%)	0.43
Heart failure (*n*, %)	4 (80%)	22 (47%)	0.35
Planned coronary angiography (*n*, %)	1 (20%)	11 (23%)	0.69
Emergency coronary angiography (n, %)	5 (100%)	35 (74%)	0.52
STEMI/NSTEMI (*n*, %)	3 (60%)	7 (15%)	**0.01**
UA (*n*, %)	2 (40%)	33 (70%)	0.39
Arterial stenosis (*n*, %)	3 (60%)	16 (34%)	0.51
Creatinine (μmol/L)	81.2 ± 26.4 80.6 (53.6–109.3)	80.9 ± 19.3 77.1 (67.8–90.2)	0.49
Baseline GFR	95.9 ± 32.39 92.8 (63.2–130.2)	82.0 ± 18.5 83.9 (73.4–93.4)	**0.08**
eGFR at admission < 60 mL/min/1.73 m^2^ (*n* %)	0 (0%)	7 (15%)	0.97
Contrast volume (ml)	140 ± 79.3	110.8 ± 89.2	0.49

Bolded values indicate statistical significance

*DM* diabetes mellitus, *CKD* chronic kidney disease, *STEMI *ST elevation myocardial infarction, *NSTEMI* non-ST-elevation myocardial infarction, *UA* unstable angina

Table 3 summarizes median levels of NGAL (ng/mL), L-FABP (ng/mL), IL-18 and F-FABP in patients who developed CI-AKI and those who did not.

**Table 3:** Comparison of selected markers levels in patients who developed CI-AKI and those who did not

Biomarker	Time (h)	Group of patients who developed CI-AKI	Group of patients who did not develop CI-AKI	*p* value
NGAL (ng/mL)	0	164.8 (136.2–309.3)	157.9 (92.4–220.2)	0.52
	24	213.6 (85.0–362.5)	208.0 (157.8–299.9)	0.44
	72	192.7 (83.5–289.8)	118.5 (73.4–173.9)	0.25
KIM-1 (pg/mL)	0	113.0 (47.9–365.9)	70.2 (62.4–311.2)	0.95
	24	232.6 (98.1–503.9)	103.7 (42.4–341.9)	0.07
	72	118.5 (74.0–157.1)	90.3 (34.3–420.4)	0.89
L-FABP (ng/mL)	0	3.7 (1.8–5.3)	4.7 (2.9–5.0)	0.98
	24	4.5 (3.2–5.5)	4.8 (2.7–5.3)	0.37
	72	5.0 (3.7–5.4)	4.6 (3.0–5.4)	0.79
IL-18 (pg/mL)	0	291.3 (271.5–380.6)	261.6 (236.8–353.3)	0.58
	24	340.9 (326.0–427.7)	264.1 (219.4–350.8)	**0.05**
	72	316.1 (291.3–358.3)	251.4 (244.2–328.5)	0.38

Bolded value indicate statistical significance

No significant differences were observed between selected markers levels in patients who developed CI-AKI and those who did not. After 24 h, only serum levels of IL-18 were much higher in patients with CI-AKI than in those without acute kidney injury, however, this difference was on the verge of significance. In case of NGAL, KIM-1 and IL-18, an increase in their serum levels can be observed after 24 h in CI-AKI group, while after 72 h, a decrease in serum concentration of these three markers can be seen. In turn, constant increase in L-FABP can be observed even after 72 h in this group. In patients without CI-AKI, an increase in NGAL and KIM-1 levels was found after 24 h and a decrease after 72 h. L-FABP and IL-18 levels seemed to be rather stable in this group in all time points. Despite a lack of statistical significance, it can be observed that serum levels of analyzed biomarkers were higher in the group of patients who developed CI-AKI.

Table 4 presents changes in studied biomarkers serum levels in all patients enrolled into this study.

**Table 4:** The comparison of concentrations of studied biomarkers at baseline, after 24 h and 72 h from the procedure in all patients included in this study

	Time (baseline)	Time (24 h)	Time (72 h)	Significance
NGAL (ng/mL)	157.9 (92.4–221.0)	201.8** (156.5–299.9)	***118.5## (73.4–198.7)	***p < 0.0001***
KIM-1 (pg/mL)	96.0 (47.9–366.0)	130.2 (42.4–341.9)	102.2 (35.0–299.1)	NS (*p* = 0.59)
IL-18 (pg/mL)	261.6 (241.7–365.7)	281.4 (226.9–350.8)	280.4 (246.7–340.9)	NS (*p* = 0.86)
L-FABP (ng/mL)	4.7 (2.7–4.9)	4.7 (2.6–5.3)	4.7 (2.9–5.4)	NS (*p* = 0.49)

Friedman test

**p < 0.01; ***p < 0.0001 vs baseline; ^##^*p* < 0.01 vs time (1 day)

The statistically significant changes in biomarkers serum levels in time were observed only in the case of NGAL (Table 5).

**Table 5:** The analysis of relationship between selected markers and the decrease in eGFR level in all patients included in this study

	Time (24 h)	Significance	Time (72 h)	Significance
NGAL (ng/mL)	79.8 (136.1–279.8)	0.76	71.4 (118.5–241.8)	0.60
KIM-1 (pg/mL)	40.3 (74.9–282.3)	0.11	34.7 (112.8–198.1)	0.53
IL-18 (pg/mL)	215.7 (291.3–358.3)	0.96	234.3 (269.0–316.1)	0.42
L-FABP (ng/mL)	2.5 (4.8–5.5)	0.32	3.4 (5.0–5.4)	0.65

No significant correlations were observed between the decrease in eGFR level and increase in creatinine level after 24 h and 72 h days from the procedure and the levels of selected biomarkers (Table 6).

**Table 6:** The analysis of relationship between selected markers and the increase in creatinine level in all patients included in this study

	Time (1 day)	Significance	Time (3 day)	Significance
NGAL (ng/mL)	80.0 (136.2–272.9)	0.79	72.0 (122.5–227.1)	0.58
KIM-1 (pg/mL)	40.4 (70.2–252.1)	**0.06**	34.8 (96.8–195.6)	0.38
IL-18 (pg/mL)	215.7 (291.3–358.3)	0.96	234.3 (269.0–316.1)	0.42
L-FABP (ng/mL)	2.5 (4.7–5.5)	0.98	2.8 (4.8–5.4)	0.98

## Discussion

Contrast-induced acute kidney injury (CI-AKI) is a cause of acute renal failure associated with in-hospital procedures [24]. The determination of serum creatinine (sCr) shows shortcomings due to the fact that its elevation is relatively late, indirectly reflects of kidney damage and it is influenced by extrarenal factors [25]. Differences in creatinine production related to age, sex, race, and weight, its secretion by renal tubular epithelium, fact that the secretion may be influenced by some drugs, compromised metabolism of Cr in AKI due to marked hypercatabolism, the dilution of creatinine in case of volume overload are among factors due to which creatinine is not a good marker of AKI [14]. Therefore, there is a need to search for new, sensitive and specific biomarkers of acute renal injury. Some molecules have been suggested to have potential to become such biomarkers. The aim of this study was to assess the levels of selected markers (NGAL, KIM-1, IL-18 and L-FABP) in patients who underwent planned or emergency coronary angiography and to examine correlation between their levels and the occurrence of CI-AKI. Our study group consisted of 52 patients who underwent either planned or emergency coronary angiography. Patients mean age was 66.4 ± 8.7 years, almost 60% of them were males, nearly 90% suffered from hypertension, one/third from diabetes mellitus, half of patients had heart failure, 17% atrial fibrillation and 13% had eGFR at admission < 60 mL/min/1.73 m^2^. Planned coronary angiography was performed in 23%, while the rest of patients underwent emergency procedure (77%). The main reason for coronary angiography was unstable angina (67%) and in 37% arterial stenosis was observed. Medium serum level of potential biomarkers at admission were as follows: NGAL—201.8 ng/mL (156.5–299.9 ng/mL), KIM-1—130.2 pg/mL (42.4–341.9 pg/mL), IL-18—261.6 pg/mL (241.7–365.7 pg/mL) and L-FABP—4.7 ng/mL (2.7–5.0 ng/mL).

Of the 52 recruited patients, 5 (9.6%) developed CI-AKI. According to literature, the strongest risk factor for CI-AKI development is the presence of pre-existing CKD [22]. In this study, patients who developed CI-AKI were more likely to be older, to have STEMI or NSTEMI and to show have higher GFR. The last observation may be surprising, but it could be associated with the fact that these patients received greater volume of contrast than patients who did not develop AKI (140 ± 79.3 vs. 110.8 ± 89.2). Moreover, all of them underwent emergency procedures, so it can be hypothesized that they were not provided with sufficient hydration before the surgery. Connolly et al. [22] demonstrated that in their study CI-AKI was considerably associated with increasing age, lower GFR, higher creatinine, higher volumes of contrast, anemia, diabetes and administration of intravenous fluids. Also in our study, the prevalence of diabetes in patients who developed CI-AKI was higher in comparison to patients without AKI (60% vs. 26%), however, the difference was not statistically significant.

In this study, we generally failed to find any significant differences between selected markers levels in patients who developed CI-AKI and those who did not. After 24 h, only serum levels of IL-18 were much higher in patients with CI-AKI than in those without acute kidney injury, however, this difference was on the verge of significance. In case of NGAL, KIM-1 and IL-18, an increase in their serum levels can be observed after 24 h in CI-AKI group, while after 72 h from the procedure a decrease in serum concentration of these three markers can be seen. In turn, constant increase in L-FABP was observed even after 72 h in CI-AKI group. In patients without CI-AKI, an increase in NGAL and KIM-1 levels was found after 24 h and a decrease after 72 h. L-FABP and IL-18 levels seemed to be rather stable in this group in all time points. Despite a lack of statistical significance, it can be observed that serum levels of analyzed biomarkers were higher in the group of patients who developed CI-AKI. According to available literature NGAL is released into plasma and urine after renal tubular cell damage, which results in an increase in its plasma and urine concentration. NGAL secretion occurs much earlier than the rise in serum concentration of creatinine [14, 26]. Therefore, NGAL seems to have potential to become a great, independent predictor of AKI [14, 27, 28]. Numerous studies indicated that NGAL could be an early, sensitive and non-invasive biomarker for predicting CI-AKI in various cardiovascular conditions [24, 29, 30]. Similar increase within 24 h followed by a decline in NGAL concentration was observed by Padhy et al. [31]. They demonstrated that serum NGAL increased sharply at 4 h after the angiography and then it gradually diminished to the level that was near normal range at 48 h in AKI cases. In that study, the optimum cut-off of serum NGAL was found to be 155.2 ng/mL at 4 h and 89.5 ng/mL at 24 h from procedure [31]. Other studies have found NGAL elevation in patients who developed CI-AKI after 2–4 h post-angiography [22, 32–34]. In turn, according to Connolly et al. [22], the best predictive time-point for measurement of plasma NGAL (> 964 ng/dL) was the 6 h. They suggested that negative result of plasma NGAL determination at 6 h meant that patients had low risk (< 4%) of developing CI-AKI and so they could be safely discharged from hospital care with appropriate CI-AKI advice. Also, the study conducted by Quintavalle et al. [34] demonstrated the use of serum and urine NGAL levels at 6 h might allow for the exclusion of CI-AKI development (serum NGAL sensitivity 74%, specificity 52%, AUC 0.62, *p* = 0.045 and urine NGAL sensitivity of 75%, specificity 52%, AUC 0.61, *p* = 0.001). A meta-analysis of 2538 patients revealed that AUC-ROC of NGAL for predicting AKI across all settings was 0.83 (95% CI, 0.74–0.91) when a median cut-off value of > 150 ng/mL and common creatinine-based definition of AKI (defined as Cr increase > 50% within 7 days) were used [4, 35, 36]. Despite numerous studies, the optimal time-point for NGAL determination following coronary angiography has not yet been well elucidated [24].

Another biomarker under our examination was L-FABP. Despite the fact that our study failed to find any significant correlation between the occurrence of CI-AKI and levels of this marker, a few other studies have suggested that L-FABP level increases between 4 and 24 h post-contrast in CI-AKI cases [22, 32, 34]. According to Connolly et al. [22], the most predictive time point for the determination of L-FABP was at 4 h (> 12.1 ng/mL) when it exhibited best sensitivity of 42.3% and specificity of 90.2%, *p* = 0.001, AUC 0.69. Their study suggests that serum L-FABP may be used alone or in the combination with plasma NGAL as a pre-procedural screening tool. Other suggested markers of AKI, such as IL-18 and KIM-1 have not been studied in detail in serum, however, the available data concerning urinary levels suggests that their increase is seen after 24–48 h following contrast [22, 37–39]. Studies performed on animal models confirmed that caspase-1-mediated production of IL-18 played a harmful role in AKI [40]. A nested case–control study, which was performed within the Acute Respiratory Distress Syndrome (ARDS) Network trial, found that urine IL-18 values 24 h before the development of AKI enabled the prediction of AKI development after the adjustment for baseline and clinical characteristics. In that study, urine IL-18 value > 100 pg/mL was associated with a 6.5-fold increased odds for developing AKI within the next 24 h (*p* = 0.0009). Moreover, the area under the ROC curve for the urine IL-18 level within the next 24 h was 73% and before 48 h was 65%, which implies its good performance for the early diagnosis of AKI [40, 41]. According to authors, immediate increase in serum IL-18 after cardiopulmonary bypass (CPB) was ascribed to the destruction of macrophages by the procedure, while the elevated levels of IL-18 after two hours was associated with systemic inflammation resulting from CPB [42, 43]. Moreover, they suggested that significantly higher serum IL-18 levels in AKI were related to ischemia, hypotension, hypoperfusion and the release of cytokines [42]. Also the study of patients who after cardiac surgery developed AKI and those who did not, demonstrated significantly higher plasma KIM-1 levels in group with AKI in comparison with healthy volunteers (*p* < 0.001) and with patients who had cardiac surgery but did not develop AKI [17]. In that study, AUC-ROC of plasma KIM-1 was 0.98 for the analysis comprising patients with established AKI versus healthy volunteers.

However, Connolly et al. [22] suggested that KIM-1 and IL-18 were not useful early CI-AKI predictors. In their study, median serum KIM-1 levels were not statistically different until 48 h post-contrast. Median KIM-1 at that time was 0.21 ng/mL in the CI-AKI group in comparison to a median of 0.16 in the non–CI-AKI group, AUC 0.65, *p* = 0.019. However, due to 48 h delay, KIM-1 exhibited no benefits over creatinine. Moreover, they revealed that serum IL-18 was not statistically significant at predicting CI-AKI at any time point, AUC range 0.40–0.54, *p* > 0.05 [22].

In this study, we observed statistically significant changes in NGAL levels in time. Considerable increase of serum NGAL concentration was seen within 24 h from contrast administration and it was followed by a marked decrease of its level. This is in agreement with the results of study performed by Shaker et al. [44], which assessed biomarkers levels in patients with normal serum creatinine undergoing coronary angiography. They observed significantly higher serum NGAL levels 4 h and 24 h after coronary interventions in comparison to the baseline value (before coronary angiography) [44]. Also Bachorzewska-Gajewska et al. [32] demonstrated considerable rise in serum NGAL after 2 and 4 h, which persisted up to 48 h after PCI.

In this study we failed to find any correlation between the level of studied markers and the decrease in eGFR or the increase in creatinine level after 24 h and 72 h. We noticed relationship between the level of KIM-1 and the increase in creatinine level, however, it failed to reach the significance. In turn, in the study performed by Sabbisetti et al. [17], plasma KIM-1 level was found to correlate with plasma creatinine (*r* = 0.58; *p* < 0.001). Malyszko et al. [45] indicated that eGFR (beta − 0.61) in multiple regression analysis was the best predictor of urinary KIM-1, and that it explained 61% of KIM-1 concentrations in renal allograft recipients. Also Tian et al. [46] found a negative correlation between plasma KIM-1 levels and the estimated glomerular filtration rate in all participants (with AKI and with CKD) (*r* = − 0.725, *p* < 0.01), which disappeared in subset analysis of AKI and CKD patients separately. In contrast to the results of our study, Dent et al. revealed that 2-h postoperative plasma NGAL levels strongly correlated with change in creatinine [47]. The discrepancies may be associated with the fact that we did not measure NGAL levels after 2 h from the procedure.

Some limitations need to be addressed. This is a small single-center study. Moreover, confounding variable such as the volume of fluid administrated before angiography was not routinely registered, therefore the analysis of its impact was not possible.

## Conclusions

Multiple serum biomarkers (NGAL, KIM-1, cystatin C, IL-18, and L-FABP) have been suggested to enable early detection of AKI before the rise in serum creatinine. Serum creatinine is still the key determinant of kidney function, however, for many reasons it is not a perfect marker. Unfortunately, our study failed to demonstrate statistically significant correlations between serum levels of selected biomarkers and the occurrence of CI-AKI. The observed trend is not sufficient for the determination of efficient AKI biomarkers. The selection of optimal biomarkers still require prospective validation in large numbers of patients with AKI and in different clinical settings. Lack of statistical significance may be associated with a small number of patients.
